# The Isothermal and Nonisothermal Crystallization Kinetics and Morphology of Solvent-Precipitated Nylon 66

**DOI:** 10.3390/polym14030442

**Published:** 2022-01-22

**Authors:** Chiah-Hsiung Tseng, Ping-Szu Tsai

**Affiliations:** Department of Chemical and Material Engineering, College of Engineering, National Kaohsiung University of Science and Technology, Kaohsiung 807618, Taiwan

**Keywords:** solvent precipitation, differential scanning calorimetry, nylon 66, crystallization kinetics

## Abstract

Solvent-precipitated nylon 66 (SP PA66) is a key material used to fabricate microfiltration membranes. The crystallization kinetics and behavior of SP PA66 were investigated through differential scanning calorimetry (DSC), polarized optical microscopy (POM) and X-ray diffraction (XRD). The Avrami equation was used to describe the isothermal crystallization of SP PA66. Nonisothermal crystallization behaviors were analyzed using Avrami equations modified by Jeziorny, Ozawa and Mo. The Avrami analysis demonstrated that the *k* values of SP PA66 were higher than those of neat PA66. The  n was between 2 and 3 indicating the presence of two- and three-dimensional mode with thermal nucleation. With an increasing cooling rate, the Jeziorny crystallization rate constant increased for SP PA66; however, the Ozawa model was not satisfactory for all SP PA66 samples. The Mo method suggested that SP PA66 had a faster crystallization rate than neat PA66 during the nonisothermal crystallization process. The solvents dissolved nylon 66, rearranged it and formed a regular hydrogen-bonded region. These regions served as nucleation sites and increased the crystallization rate constant in the subsequent melting process. The crystal morphology of the SP PA66 under the POM investigation exhibited Maltese cross spherulites. The sizes of the spherulites of SP PA66 were significantly smaller than those of neat PA66. Wide-angle XRD revealed that SP PA66 had the same crystal structure and a higher crystal perfection than neat PA66.

## 1. Introduction

Semicrystalline nylon 66 or poly(hexamethylene adipamide), which was first prepared by Carothers in 1936 [1], has been widely used in many engineering applications, including in the production of textile fibers, films, flexible packaging materials for food, electronics (e.g., connectors, surface mount devices and reflectors) and plastics for use in the automotive industry [2]; this is because of its excellent dimensional stability, mechanical strength at high temperature, chemical resistance and other advantages. Nylon 66 has high tensile strength, high thermal stability and biocompatibility. Moreover, the hydrophilicity of nylon 66 is higher than that of many others polymers because of the presence of amide groups in the polymer backbone; nylon 66 is synthesized through precipitation by various aqueous solvent solutions [3,4,5,6]. Nylon 66 membranes are extensively employed in various separation processes involving electrospun nanofibers that are applied in water and air filtration, blood purification, biosensors, tissue engineering and drug delivery, enzyme immobilization and acoustical damping [7,8,9,10,11]. Among various techniques used to prepare nylon 66 membranes, immersion precipitation is the most widely used. In this technique, a polymer is cast as a thin film and subsequently immersed in a nonsolvent bath to induce diffusion-controlled phase separation. The properties of a semicrystalline polymer are dependent on the crystalline structure formed during processing. Khanna [12,13,14,15] reported that the processing history (e.g., melt extrusion, freeze grinding or solution precipitation) leads to a memory effect that in turn enhances the crystallization rate. Muellerleile [16] hypothesized that the dissolution of a polymer in different solutions causes the disruption of the disorder of hydrogen bonds, resulting in the formation of ordered hydrogen bond regions (nucleation centers). The overall crystallization rate was faster in solvent treatment, and the mechanism underlying the crystallization of a minor crystal was completely different from that of a major crystal. The minor crystal was heterogeneously nucleated and highly constricted in the later growth period. Few studies have examined the role of solvent precipitation and the nucleating mechanism [17,18,19]. In this study, differential scanning calorimetry (DSC) was employed to investigate isothermal and nonisothermal crystallization by solvent-precipitated nylon 66 (SP PA66). Several kinetics methods were adopted to analyze its isothermal and nonisothermal crystallization processes. For complex systems, many modules have been applied to it, and good results have been obtained. Gadomski used the stochastic exponential kinetics to explain the diffusion–migration phenomenon of some (bio)polymeric complex systems and the wave-like late temporal behavior of protein aggregation [20,21]. Crystallization kinetics’ parameters based on the isothermal crystallization were analyzed using the Avrami equation. Practical processes, namely, extrusion, molding and fiber or film formation, were usually performed under dynamic and nonisothermal crystallization conditions. The nonisothermal crystallization process was quantitatively examined to optimize processing conditions in an industrial application and improve properties. Avrami equations modified by the Jeziorny, Ozawa and Mo methods were used to investigate the nonisothermal crystallization kinetics of SP PA66. Moreover, the Arrhenius and Kissinger methods were employed to determine the activation energy of the isothermal and nonisothermal crystallization of SP PA66. The structure and spherulite morphology of SP PA66 were examined through wide-angle X-ray diffraction (XRD) and polarized optical microscopy (POM), respectively. We found that the different crystallization rates can be obtained by using different solvents, so manipulating solvents and process parameters was easily achieved to obtain the desired fiber morphology, mechanical strength, shape and porosity in the application field of electrospinning. In the membrane applications, different solvent precipitations create different asymmetric structures, which exhibit different dense top layers and porous sublayers, with different water absorption, porosity and fluid flux.

## 2. Experimental Section

### 2.1. Material and Sample Preparation

Neat nylon 66 pellets used in this study were kindly provided by Grand Pacific Petrochemical Co. Ltd. (Kaohsiung, Taiwan) as polymer granules with a relative viscosity of 45.6 in formic acid (90%) at 25 °C as determined using a viscosimeter; the glass-transition temperature ($T_{g}$) and melting temperature ($T_{m}$) of granules were 56 °C and 262 °C, respectively. The sample was utilized as received. Virgin polymers were dissolved into solutions, each of which was precipitated by slowly adding an excess of stirred distilled water or ether. The samples were hot-filtered using filter paper, washed with distilled water and then dried at 80 °C for 24 h in a vacuum oven. Nylon 66 pellets were treated with 90% formic acid, 98% sulfuric acid and 100% *m*-cresol, abbreviated as PA66-FA, PA66-HS and PA66-MC, respectively. The sample containing 100 ppm CaF_2_, a nucleating agent, was abbreviated as PA66-NU, and the base polymer without CaF_2_ was abbreviated as PA66-BP.

### 2.2. Differential Scanning Calorimetry (DSC)

A Perkin-Elmer DSC-7 (MA, USA) was used for the calorimetric investigations of the melting behavior and crystallization kinetics. All DSC measurements were taken under a nitrogen atmosphere with flow rate of 20 mL·min^−1^ and calibrated using high purity indium; the weight of the samples was between 3 and 5 mg.

### 2.3. Isothermal and Nonisothermal Crystallization Process

In isothermal crystallization kinetic studies, the samples were hermetically sealed in a standard aluminum pan and heated quickly from 15 °C to 300 °C at 80 °C·min^−1^ above the melting temperature *T_m_*. The thermal history of the samples was erased by heating them at 300 °C for 10 min, and quenched at −80 °C·min^−1^ from 300 °C to the desired crystallization temperatures *T_c_*, in the range of 219−231 °C for 30 min. Enthalpy during the isothermal crystallization was recorded at different *T_c_*. After crystallization, the samples were heated to 300 °C at a rate of 10 °C·min^−1^, and the enthalpy fusion (Δ*H_f_*) was calculated from the maximum point of the curve and the area under the endothermic peak. Nonisothermal crystallization was performed as follows. The samples were rapidly heated from 15 °C to 300 °C at 80 °C·min^−1^, the samples were maintained at 300 °C for 10 min to erase their thermal history. Subsequently, the sample were cooled to 15 °C at the cooling rates of 2.5, 5, 10, 20 and 40 °C·min^−1^. The exothermal curves of heat flow as a function of temperature were recorded.

### 2.4. Polarized Optical Microscopy (POM)

The crystal morphology of all PA66 samples was measured using a Nikon LV100ND (Tokyo, Japan) polarized optical microscope equipped with a DS-Fi3 camera system and LINKAM LTS420 (Surry, UK), a hot-stage. The samples were sandwiched and melted between two glass slides, heated to 30 °C about their melting point for 5 min, and then cooled from the melt at 10 °C/min^−1^ to 220 °C while spherulites appeared and grew. The images of the samples were captured after complete crystallization by using camera.

### 2.5. X-ray Diffraction (XRD)

The crystal structure of all PA66 samples were recorded using an X-ray diffractometer system (Rigaku, D/MAX, Tokyo, Japan) with $\mathrm{Cu}-K_{\alpha }$ radiation (λ = 0.154 nm) at ambient temperature, and operated at 40 kV and 200 mA. The 2θ ranged from 0° to 50°, and the scanning rate was 5° min^−1^.

## 3. Results and Discussion

### 3.1. Isothermal Crystallization Kinetics from Avrami Equation

**Figure 1:**
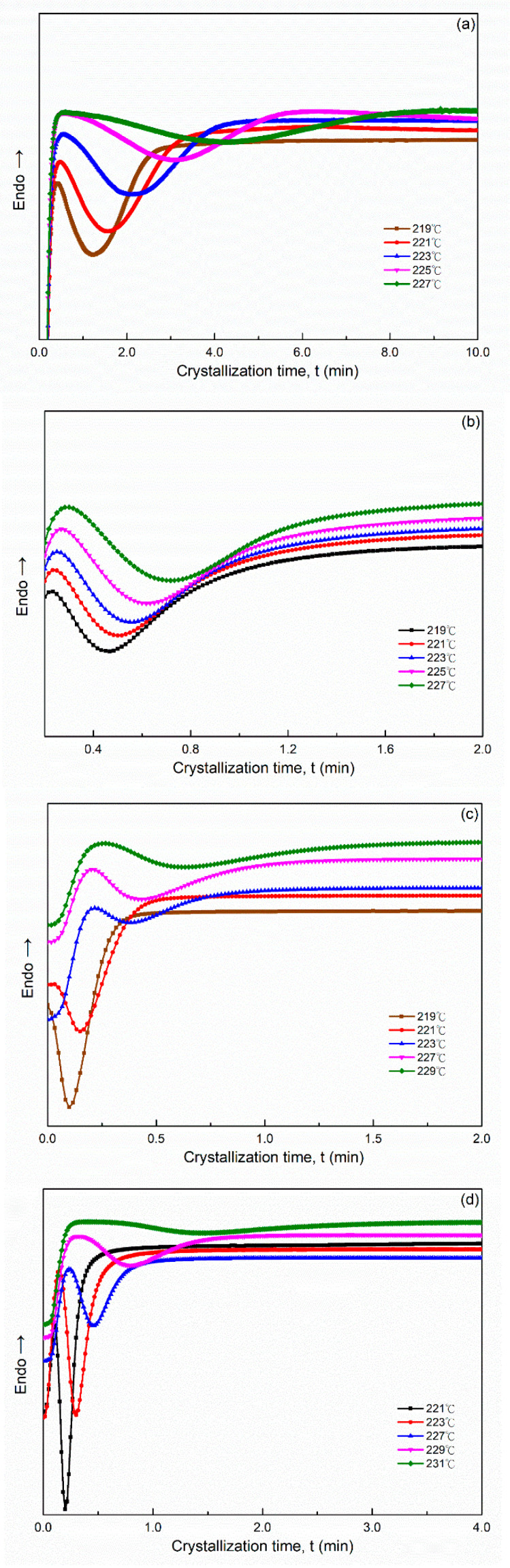

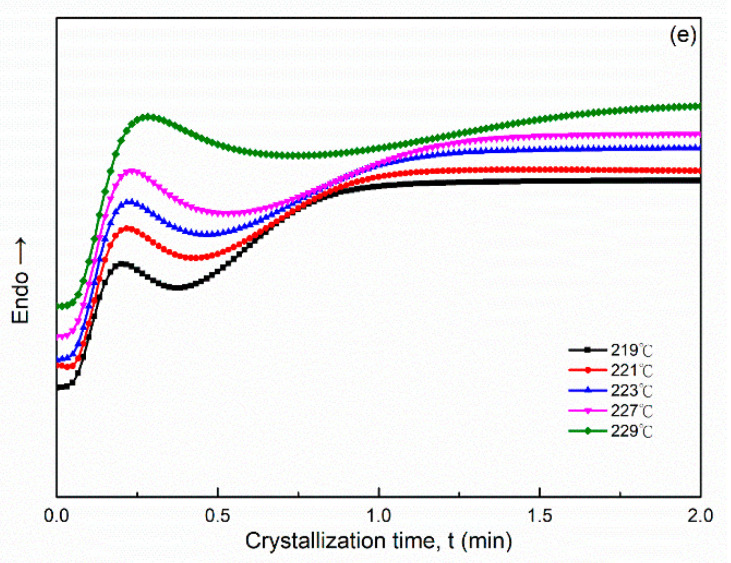
Heat flow versus time during isothermal crystallization of (**a**) PA66-BP, (**b**) PA66-NU, (**c**) PA66-FA, (**d**) PA66-HS and (**e**) PA66-MC at the different crystallization temperatures by DSC.

The effect of the solvents on the isothermal crystallization kinetics of all PA66 samples were investigated through DSC at different ranges of isothermal temperature (219–227 °C for PA66-BP and PA66-NU, 219–229 °C for PA66-FA, 221–231 °C for PA66-HS and 219–231 °C for PA66-MC). Figure 1 presents the DSC curves of the samples. For each sample, the chosen crystallization temperature was determined by performing a series of experiments at various crystallization temperatures. With increasing crystallization, in order to appear the crystallization peaks of SP PA66 samples required a longer time and became flatter, and the peak values became smaller (Figure 1). This finding indicated that the crystallization rate decreased and the SP PA66 samples required a longer time to achieve complete crystallization at a higher crystallization temperature. Because of a decrease in the supercooling temperature (Δ*T =* $T_{m}^{0}$*−T_c_*), the crystallization rate decreased with an increase in the crystallization temperature, the crystallization time increased with a decrease in the crystallization rate [22].

To study the isothermal crystallization kinetics, the relative crystallinity *X(t)* was determined using the follow equation

(1) $$X\left(t\right)=\frac{Q_{t}}{Q_{\infty }}=\frac{\int_{t_{0}}^{t_{c}}\;\left(\frac{dH_{c}}{dt}\right)dt}{\int_{t_{0}}^{t_{\infty }}\left(\frac{dH_{c}}{dt}\right)dt}\;$$

where *dH_c_* denotes the measured enthalpy of crystallization during the time interval *dt*; $Q_{t}$ is the heat generated at time *t*; $Q_{\infty }$ is the total heat generated up to the final crystallization process; *t*_0_ denotes the initial crystallization time; *t_c_* and *t*_∞_ denote the time elapsed during the course of crystallization and after the completion of the crystallization process, respectively.

The crystallization process was highly dependent on temperature. To better investigate the isothermal crystallization kinetics of PA66 polymers, the classical Avrami equation [23,24,25] presented below was used

(2) $$X\left(t\right)=1-exp\left(-kt^{n}\right)$$

Equation (2) is often written in a double logarithmic form as follows

(3) log{−ln[1−X(t)]}=logk+nlogt 

**Figure 2:**
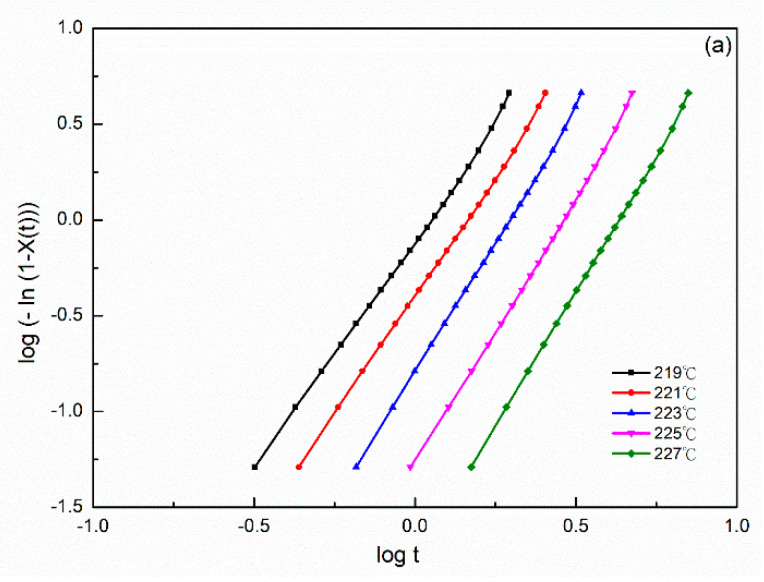

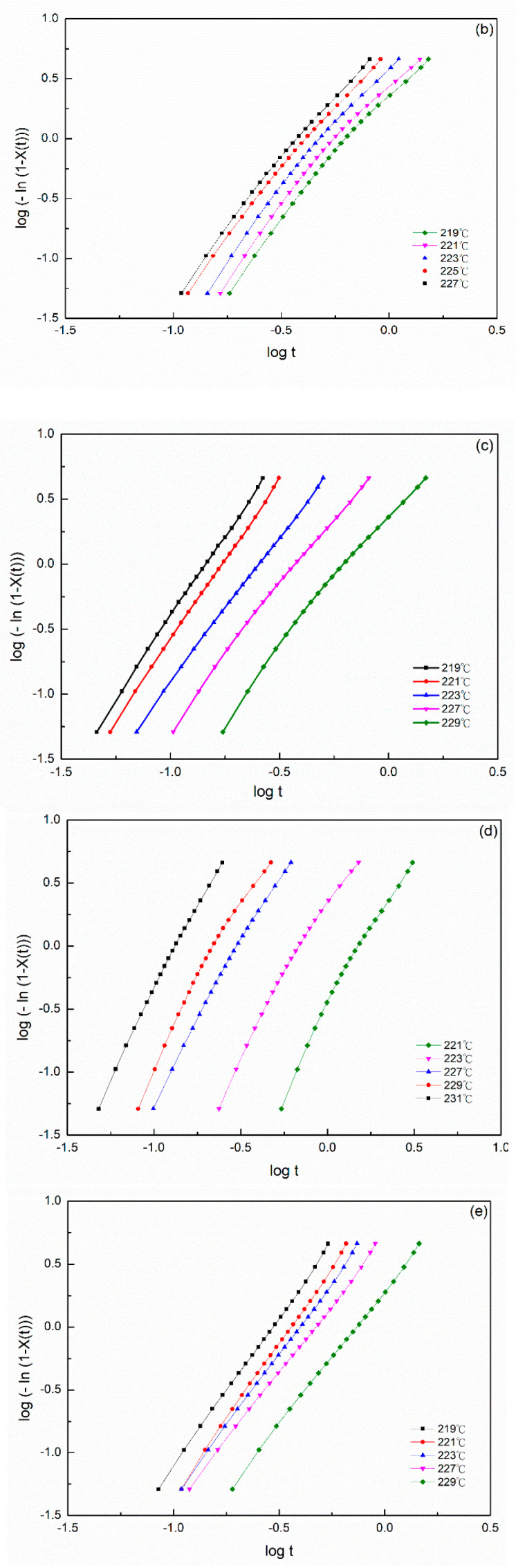
Plots of log{−ln[1−x(t)]} versus logt at the indicated temperature for isothermal crystallization of (**a**) PA66-BP, (**b**) PA66-NU, (**c**) PA66-FA, (**d**) PA66-HS and (**e**) PA66-MC.

**Figure 3:**
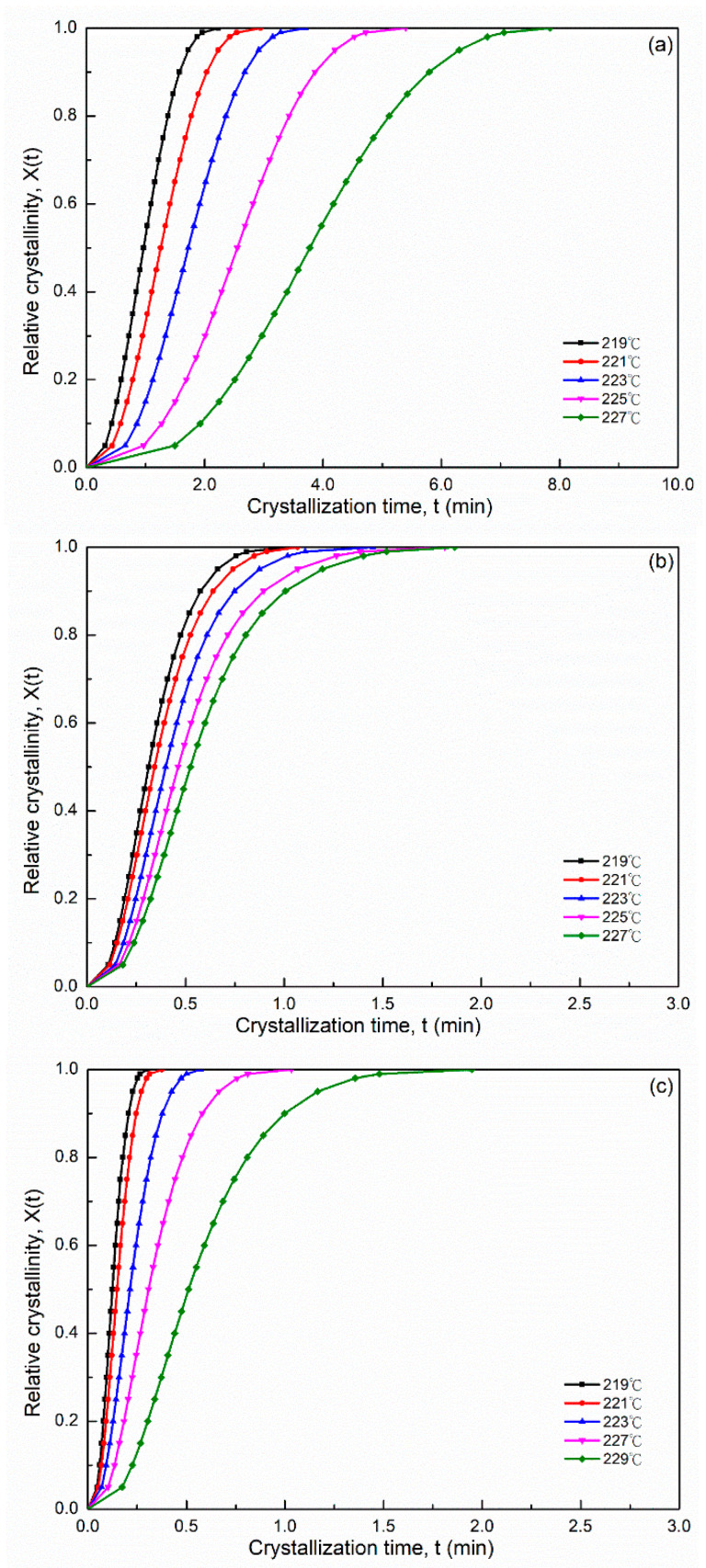

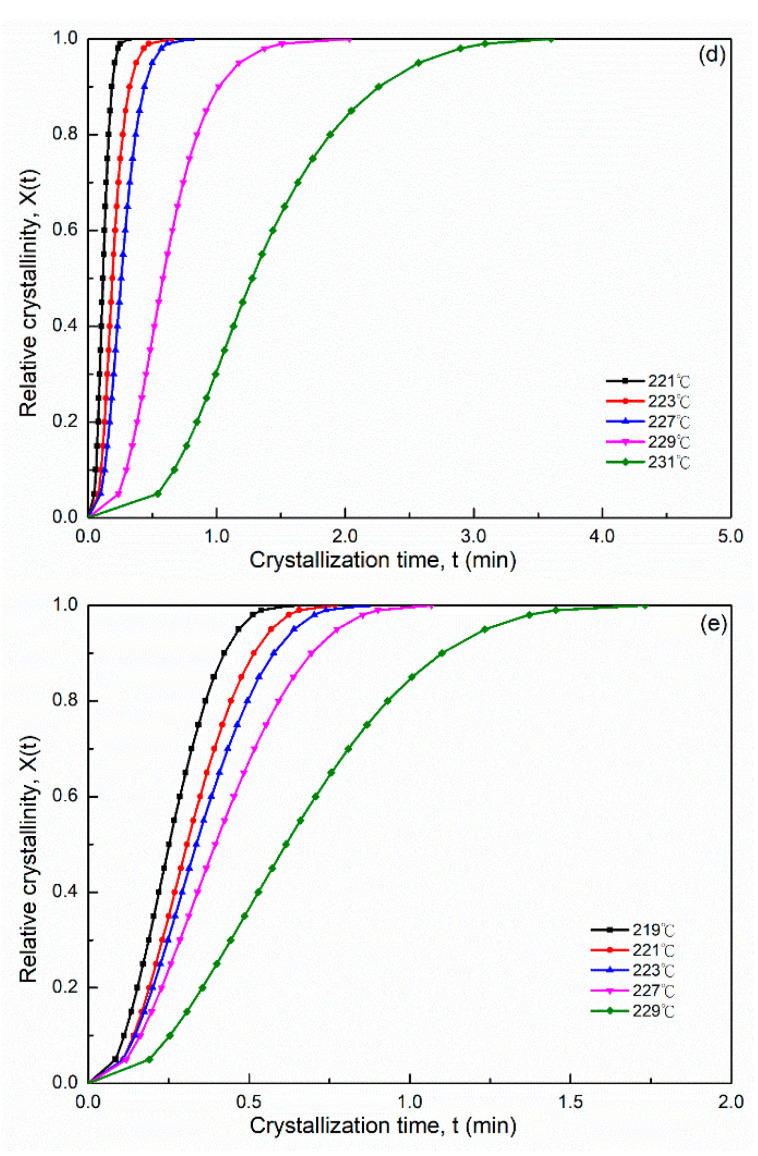
Relative crystallinity X(t) versus different crystallization time t for isothermal crystallization of (**a**) PA66-BP, (**b**) PA66-NU, (**c**) PA66-FA, (**d**) PA66-HS and (**e**) PA66-MC.

This equation was used to evaluate the Avrami parameters, where *n* is the Avrami exponent, reflecting the crystallization mechanism, *t* is the time and *k* is the kinetics rate constant involving both crystal growth and nucleation parameters. Usually, the values of *n* should be an integer between 1 and 4 for different crystallization mechanisms [26]. An Avrami parameter of *n* = 1, 2 and 3 indicated one-, two- and three-dimensional crystal growths, respectively. However, Avrami exponent *n* was not a simple integer when the other complex factors were possibly involved, including an irregular boundary of the spherulites and/or the competition of diffusion-controlled growth. The plots of log{*−*ln[1*−X(t)*]} versus log *t* exhibited a linear relationship in all PA66 samples (Figure 2). The relative degree of crystallinity was determined as a function of the crystallization time. Accordingly, the values of Avrami exponent n and the isothermal crystallization parameter k values could be obtained from the slope and intercept of the linear portion shown in Figure 2, and the results of the samples are summarized in Table 1. The values of n were between 2 and 3 for each PA66 sample. Hence, the PA66 sample might be the mixture with a crystallization mode of two-dimensional and three-dimensional with thermal nucleation [27,28], indicating that spherulites nucleated and grew freely. We obtained non-integral *n* values possibly due to the presence of crystalline branching and/or two-stage crystal growth during the crystallization process and/or mixed growth and nucleation mechanisms. The values of the crystallization rate parameters *k* of all the samples increased with a decrease in the crystallization temperature *T_c_* (Table 1). This finding indicated that the rate of nucleation and crystal growth decreased with increasing *T_c_*, and the values of *k* exhibited very different temperature-dependency characteristics. A large *k* value corresponded to a higher crystallization rate. This finding is consistent with the result of the crystallization time observed in the isothermal crystallization behavior analysis. The *k* values of the SP PA66 and PA66-NU were higher than that of the neat PA66-BP, and the PA66-HS sample was the highest at the same temperature. Another crucial parameter was the crystallization half-time $t_{1/2}$, which is defined as the time at which the extent of crystallization was 50% of the relative crystallinity and was using the following equation (Figure 3)

(4) $$t_{1/2}={\left[\frac{\ln2}{k}\right]}^{\frac{1}{n}}\;\;$$

The rate of crystallization G was the reciprocal of $t_{1/2}$; $G=\tau_{1/2}={\left(t_{1/2}\right)}^{-1}$. The values of $t_{1/2}$ and $\tau_{1/2}$ are listed in Table 1. The $t_{1/2}$ value increased with increasing *T_c_* for each sample. The values of the SP PA66 and PA66-NU were lower than the neat PA66-BP, and PA66-HS was the lowest at the same crystallization temperature. The data indicated that the solvent precipitation significantly accelerated nylon 66 crystallization processes. The parameter $t_{max}$ indicated the time required to achieve the maximum crystallization rate. Because $t_{max}$ corresponded to the point at which dQ(t)/dt=0, the *Q(t)* was defined as follows:

(5) $$t_{max}={\left(\frac{n-1}{nk}\right)}^{1/n}\;$$

As shown in Figure 1, the $t_{max}$ of the heat flow curves was obtained using Equation (5). The $t_{max}$ of the SP PA66 sample was lower than that of the neat PA66-BP sample (Table 1). Figure 3 shows the relation curves between the crystallization time and relative crystallinity X(t) for the samples at different crystallization temperatures. As shown in Figure 3, characteristic sigmoid isothermals shifted to the right with an increase in crystallization temperature. The relative crystallinity decreased with the increasing crystallization temperature *T_c_* at a given crystallization time (Table 1). The flat part of the sigmoid curves was considered to generally represent the secondary crystallization step caused by spherulite impingement in the later stage of crystal growth.

### 3.2. Isothermal Crystallization Activation Energy (*Δ*E)

**Figure 4:**
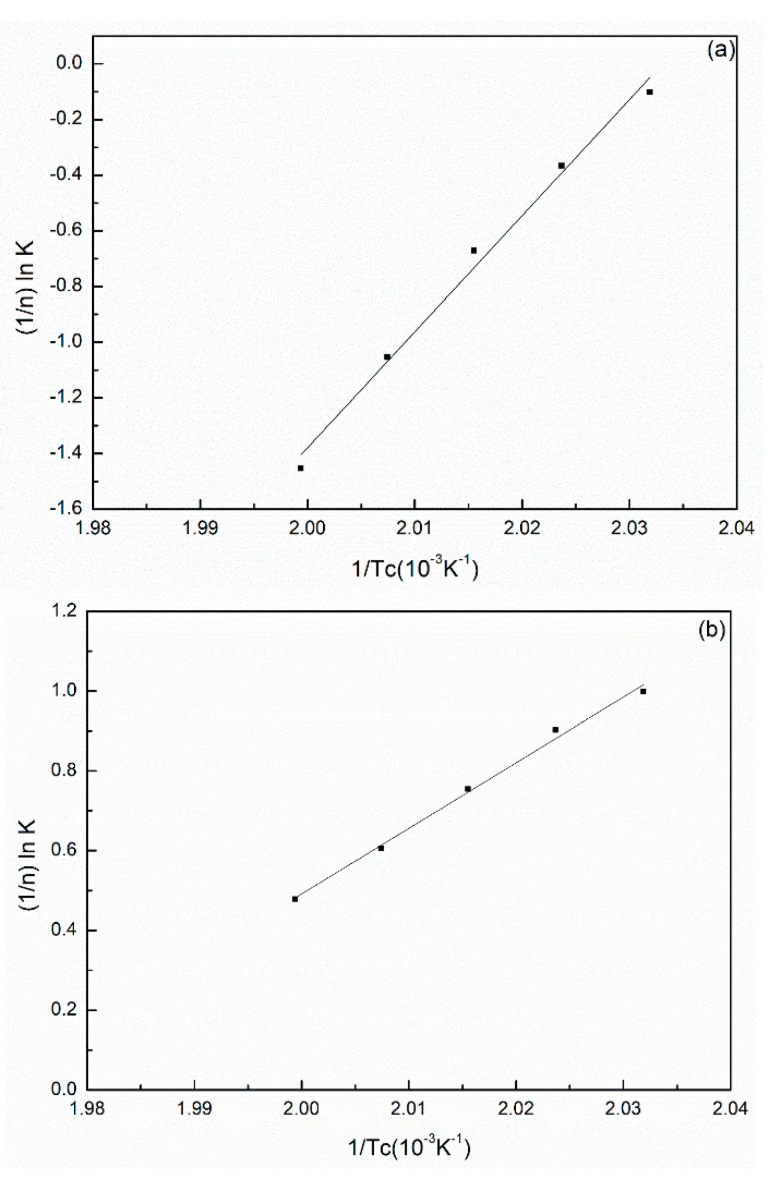

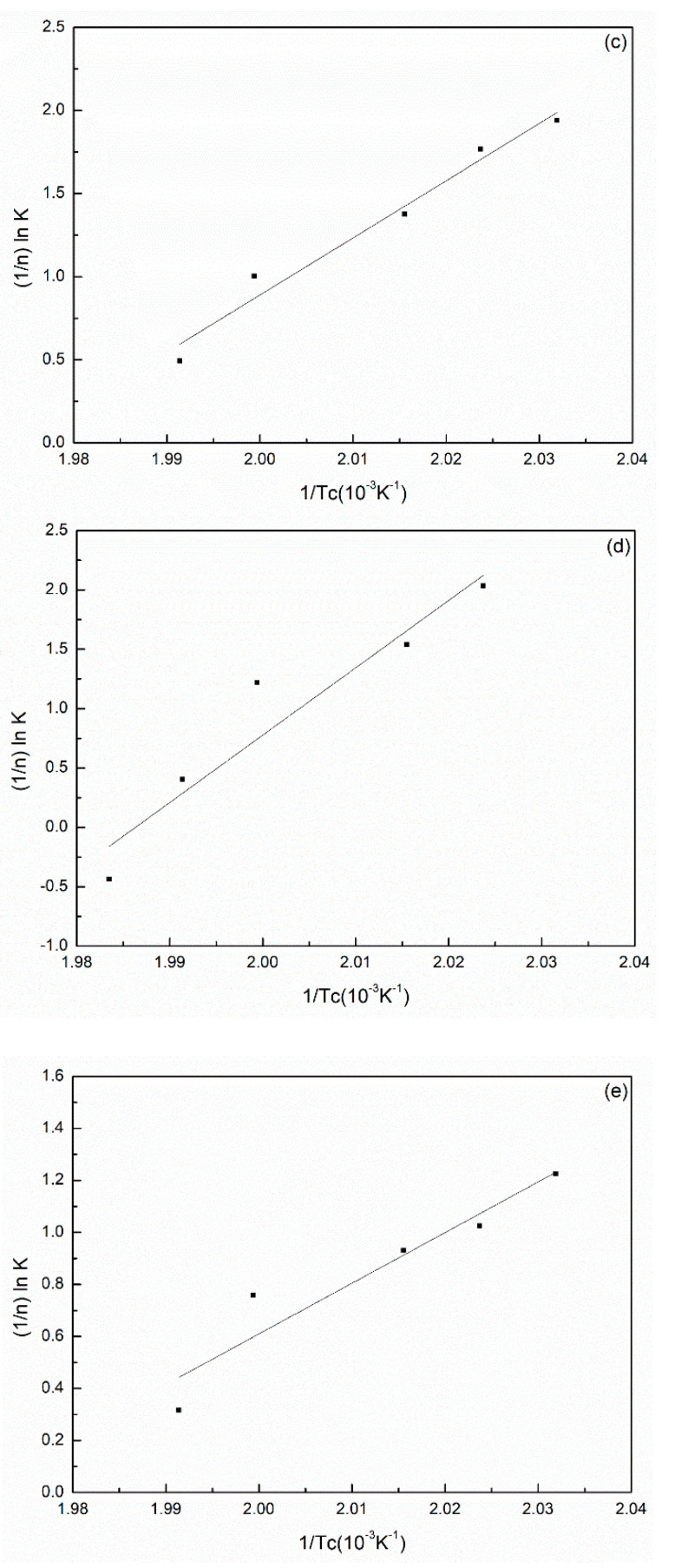
(1/n)lnk versus $1/T_{c}$ for Avrami parameter k deduced from isothermal crystallization of (**a**) PA66-BP, (**b**) PA66-NU, (**c**) PA66-FA, (**d**) PA66-HS and (**e**) PA66-MC.

The approximate activation energy and crystallization rate constant of the PA66 samples could be determined by the following Arrhenius equation, as shown in Equations (6) and (7) [29]

(6) $$K^{1/n}=k_{0}exp\left(-\frac{\Delta E}{RT_{C}}\right)\;\;\;\;\;\;\;\;\;\;\;\;\;\;\;\;\;\;\;\;\;\;\;\;\;\;\;\;\;\;\;\;\;\;\;\;\;$$

(7) $$\frac{1}{n}\ln K=\ln k_{0}-\frac{\Delta E}{RT_{C}}\;\;\;\;\;\;\;\;\;\;\;\;\;\;\;\;\;\;\;\;\;\;\;\;\;\;\;\;\;\;\;\;\;\;\;\;\;$$

where *k*_0_ is a constant independent of temperature, *n* is the Avrami index, Δ*E* is the crystallization activation energy(kJ/mol), *T_c_* is the absolute temperature of isothermal crystallization and *R* is the gas constant. Δ*E* was determined from the slope coefficient of the plots of (1/n)lnK versus 1/*T_c_*_,_ and exhibited a straight line in Figure 4. The activation energy values were −285.72, −162,13 and −457.62 kJ/mol for the PA66-FC, PA66-MC and PA66-HS, respectively, for the nonisothermal process; these values were higher than that of the neat PA66 sample (−486.45 kJ/mol) [30]. In the isothermal crystallization process, the activation energy was strongly dependent on the type of solvent.

**Figure 5:**
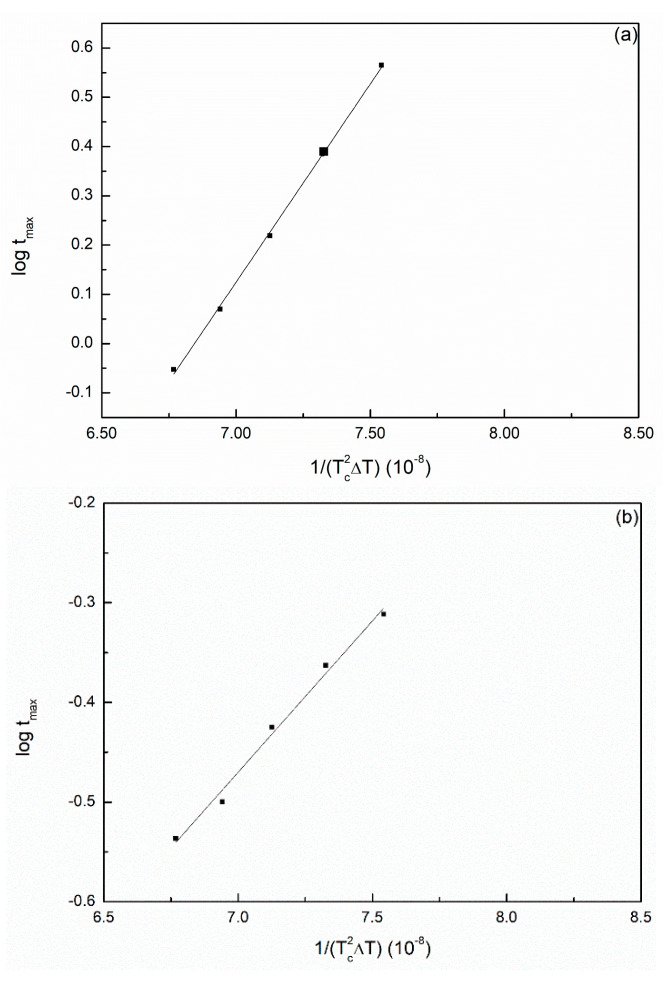

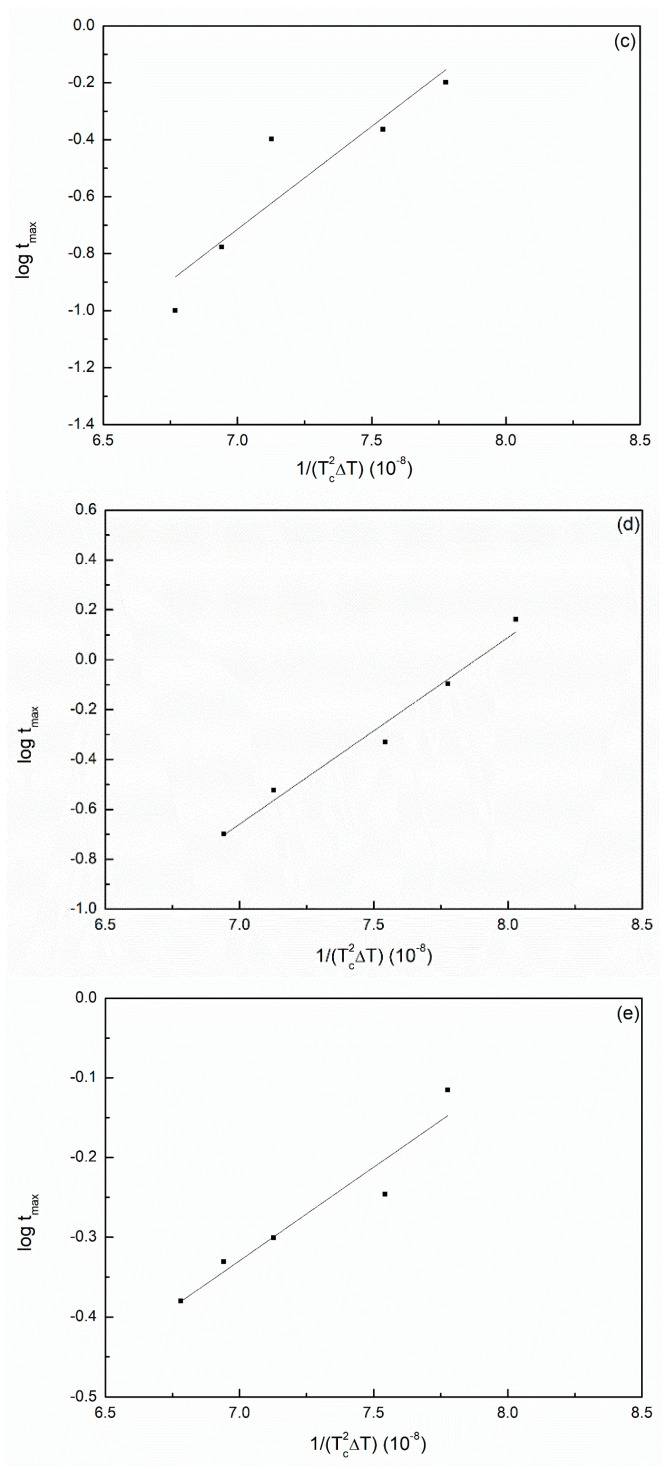
Plots of $\log\;t_{max}$ versus $1/T_{c}^{2}\Delta T$ of isothermal crystallization of (**a**) PA66-BP, (**b**) PA66-NU, (**c**) PA66-FA, (**d**) PA66-HS and (**e**) PA66-MC.

The Turnbull–Fisher equation is as follows [31]

(8) $$\ln G=\ln G_{0}-\frac{\Delta E^{*}}{kT_{c}}-\frac{\Delta F^{*}}{kT_{c}}$$

where $G_{0}$ is a preexponential factor, G is the spherulitic growth rate, *T_c_* is the crystallization temperature, *k* is the Boltzmann constant, ΔE*, which was transported as a chain segment from the supercooled state to the crystalline phase, is the free energy of activation and ΔF* is the free energy of the formation of a nucleus of critical size. The nucleation term ΔF*/kT_c_ rapidly became dominant for the crystallization rate at high temperature. Hence, the crystallization temperature approached the melting temperature. In the current study, the $T_{c}$ value ranged from 219 °C to 231 °C and approached *T_m_* = 262 °C. Therefore, the Equation (8) could be written as follows:

(9) $$\ln G=\ln G_{0}-\Delta F^{*}/kT_{c}\;$$

where the crystallization rate was controlled by a single nucleation term. The term for ΔF*/kT_c_ was adapted as derived by Hoffmann [32,33,34], the expression can be represented as follows

(10) $$\ln G=\ln\;G_{0}-\frac{xT_{m}^{0}}{T_{c}^{2}\left(T_{m}^{0}-T_{c}\right)}\;\;$$

where $T_{m}^{0}$ is the equilibrium melting point ($T_{m}^{0}$ = 280 °C) [28], and *x* is a parameter related to the heat of fusion and interfacial free energy. The following Equation (8) could be obtained from Equations (3), (5) and (10) [35]

(11) $$\;\log\;t_{max}=B-\frac{C}{2.303\cdot T_{c}^{2}\cdot \Delta T}\;$$

where Δ*T* is the degree of supercooling (ΔT = $T_{m}^{0}-T_{c}$) and B and C are constants. Equation (11) was used to evaluate whether the PA66 samples can be examined using the Avrami equation; if a plot of $\log\;t_{max}$ versus $1/T_{c}^{2}\Delta T$ is a straight line, SP PA66 would follow primary crystallization at $t_{max}$. The plots for the SP PA66 sample exhibited a good linear relationship (Figure 5).

### 3.3. Nonisothermal Crystallization Behaviors

#### 3.3.1. Modified Avrami Equation by Jeziorny

**Figure 6:**
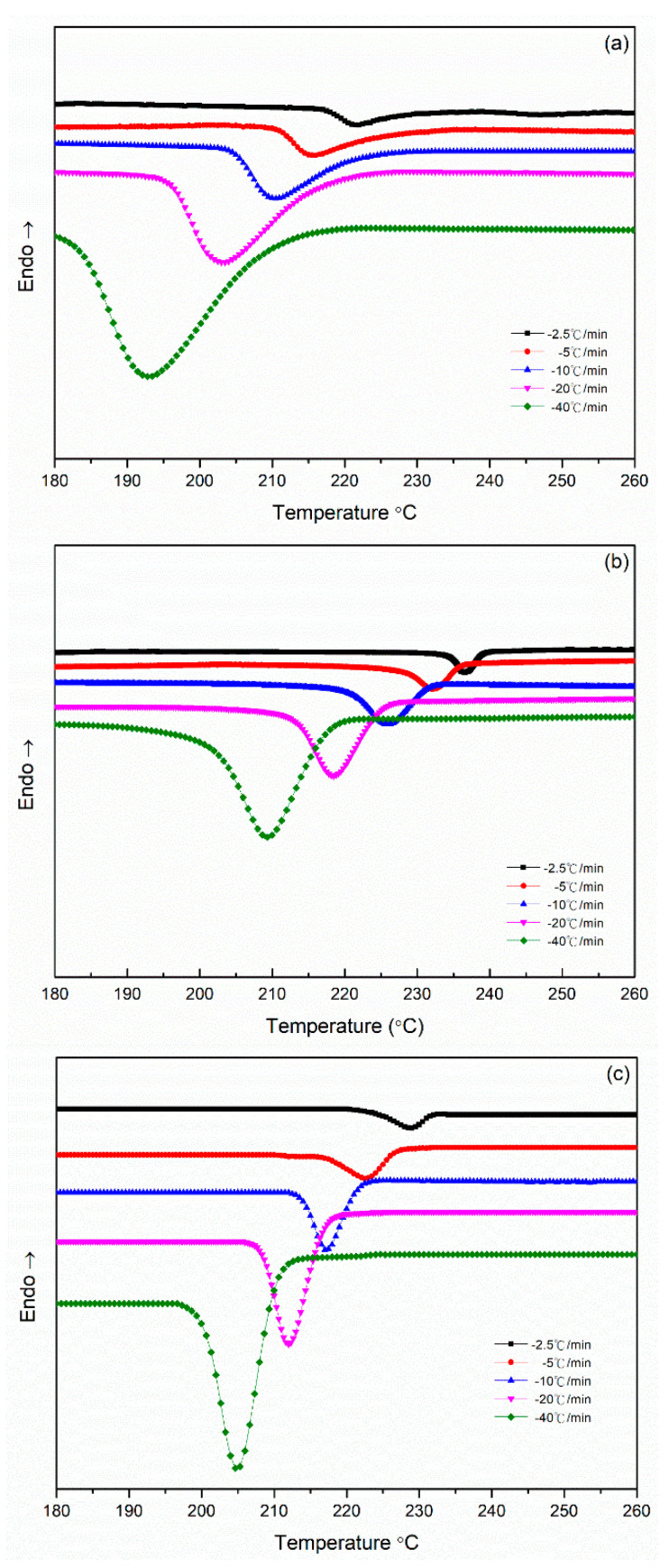

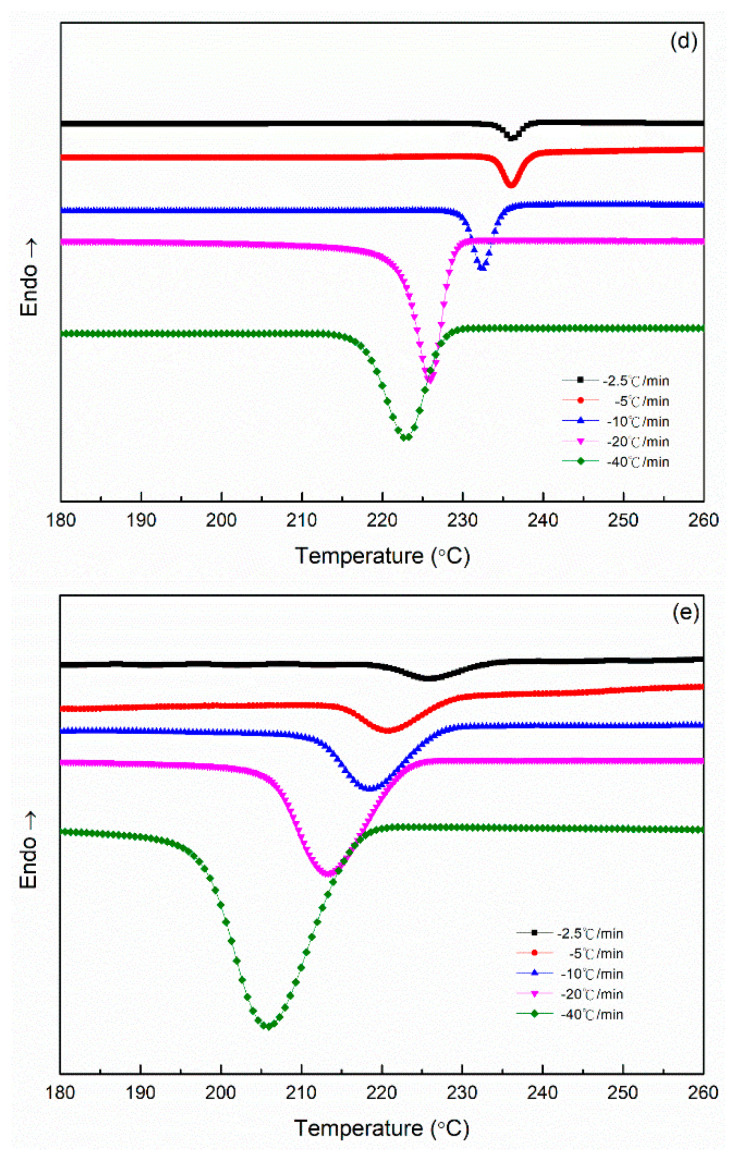
Heat flow versus temperature during nonisothermal crystallization of (**a**) PA66-BP, (**b**) PA66-NU, (**c**) PA66-FA, (**d**) PA66-HS and (**e**) PA66-MC at different cooling rates.

**Figure 7:**
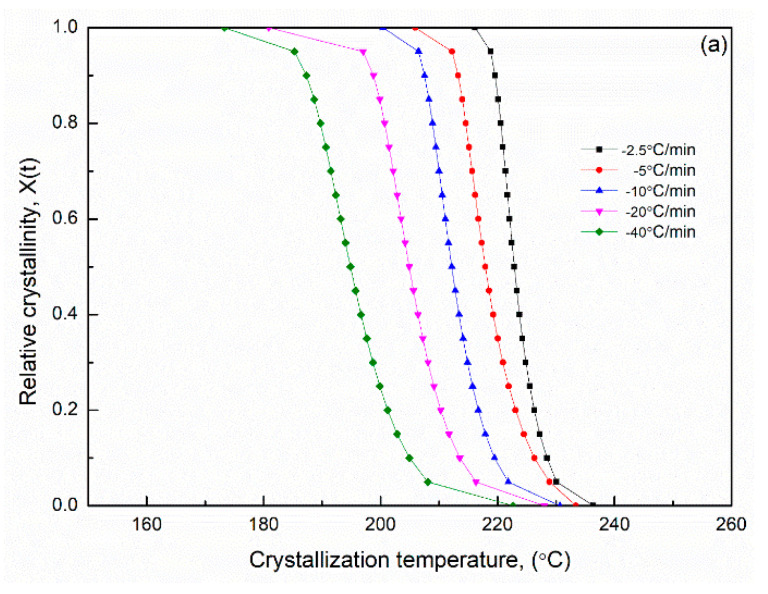

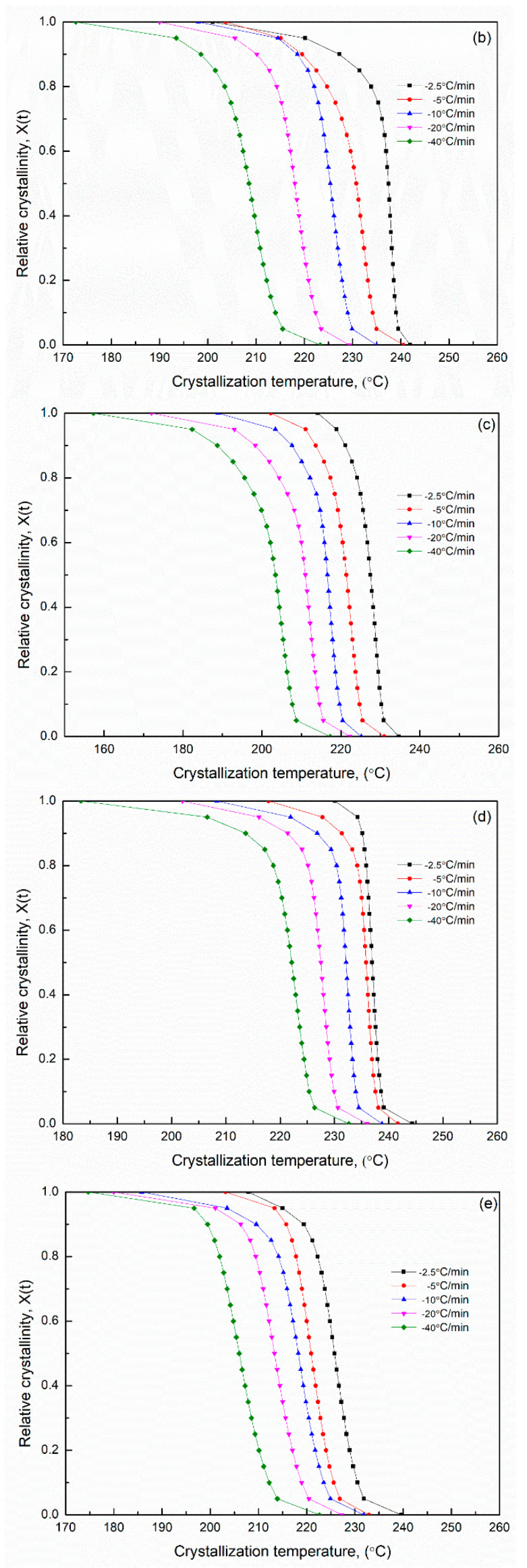
Relative crystallinity X(t) at different crystallization temperatures *T* in the process of nonisothermal crystallization of (**a**) PA66-BP, (**b**) PA66-NU, (**c**) PA66-FA, (**d**) PA66-HS and (**e**) PA66-MC.

Figure 6 presents the nonisothermal crystallization exothermic peaks of the PA66 samples obtained under different cooling rate *Φ*. T* was the peak temperature when the crystallization rate was the maximum, and T* shifted to a lower temperature region and became broader with an increase in the cooling rate (Figure 6). The peak temperature T* and the crystallization peak times $t_{max}$ and its relative crystallinity at different cooling rates are listed in Table 2. For the maximum crystallization temperature (or time), different rate dependencies were observed in the melt crystallization of the SP PA66 sample. The relative crystallinity *X*(*t*) at different cooling rates obtained through DCS is shown in Figure 7. A series of reversed *S*-shaped curves were obtained because of the spherulites impingement in the final crystallization stage; the curves tended to flatten. The value of *T* (Figure 7) was transformed to crystallization *t* as follows

(12) $$t=\frac{\left|T_{0}-T\right|}{\Phi }$$

**Figure 8:**
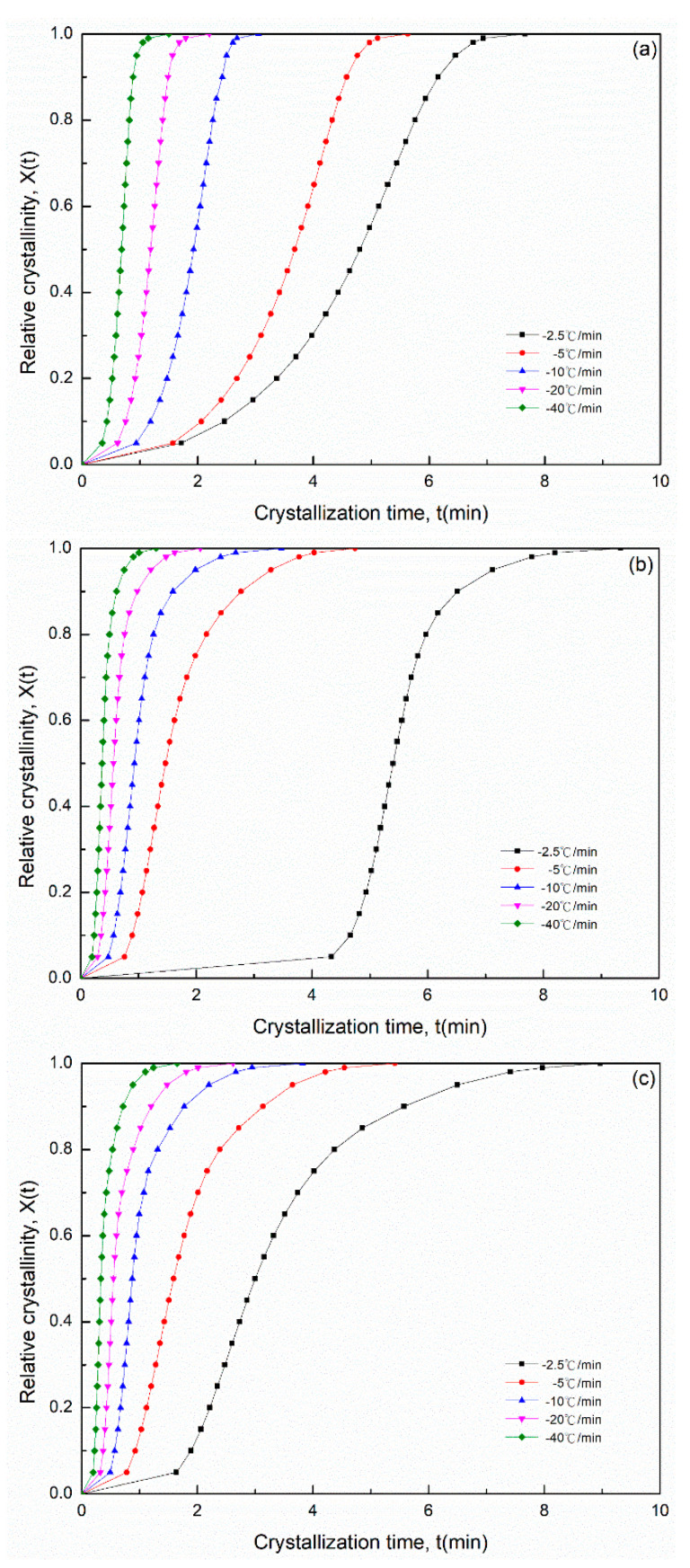

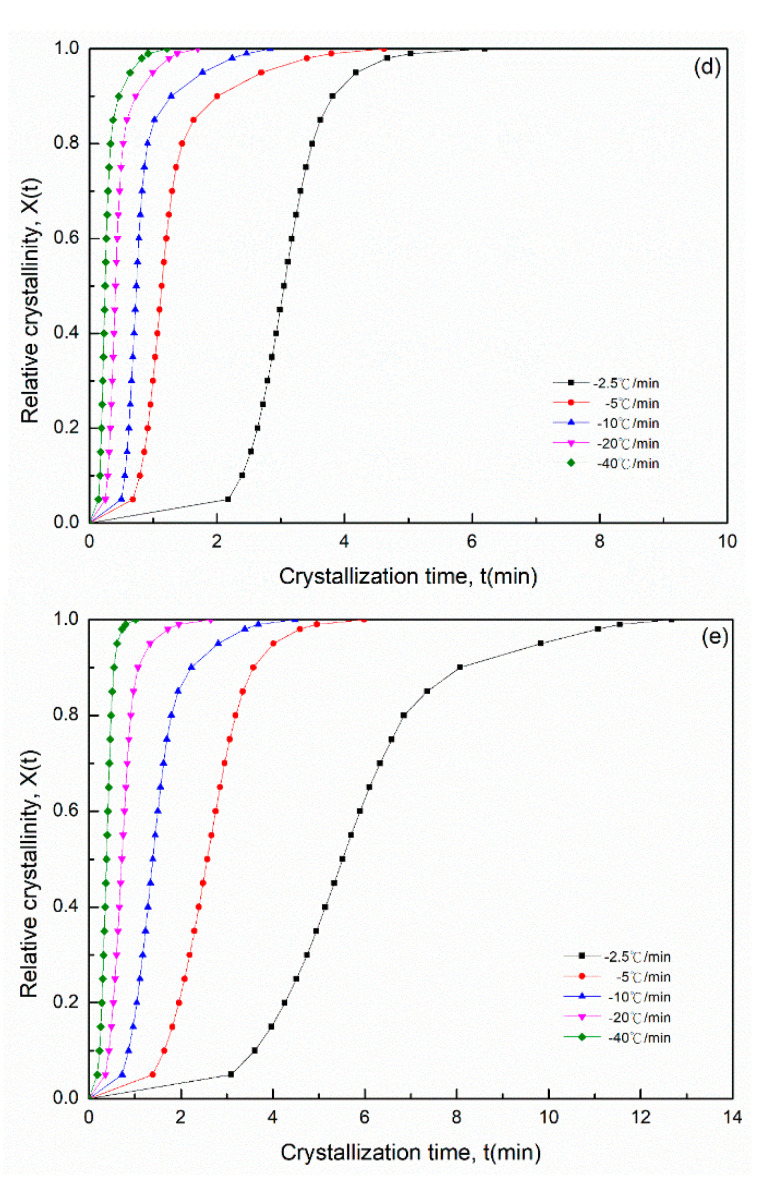
Relative crystallinity X(t) at different crystallization times *t* in the process of nonisothermal crystallization of (**a**) PA66-BP, (**b**) PA66-NU, (**c**) PA66-FA, (**d**) PA66-HS and (**e**) PA66-MC.

The plots of crystallization *t* on the *x*-axis are shown in Figure 8. All the curves of the plots had similar sigmoidal shapes. We obtained the values of *T* or *t* at the different cooling rates from Figure 7 and Figure 8 at a given relative crystallinity *X*(*t*). On the basis of the assumption that the crystallization temperature is constant, Mandelkern [36] assumed that the primary stage of nonisothermal crystallization can be described using the Avrami equation as follows

(13) $$\;1-X\left(t\right)=exp\left[-Z_{t}t^{n}\right]$$

(14) $$\log\left\{-\ln\left[1-X\left(t\right)\right]\right\}=n\;\log\;t+\log Z_{t}$$

where $Z_{t}$ is the rate constant involving both nucleation and growth rate parameters and *n* is the Avrami exponent in the nonisothermal crystallization process. To use the equation for analyzing nonisothermal crystallization behavior, Jeziorny [37] modified the crystallization rate $Z_{t}$ in the Avrami equation through division by cooling rate *Φ* to incorporate the temperature change during the nonisothermal crystallization process, as follows:

(15) $$\log\;Z_{c}=\frac{\log Z_{t}}{\Phi }$$

**Figure 9:**
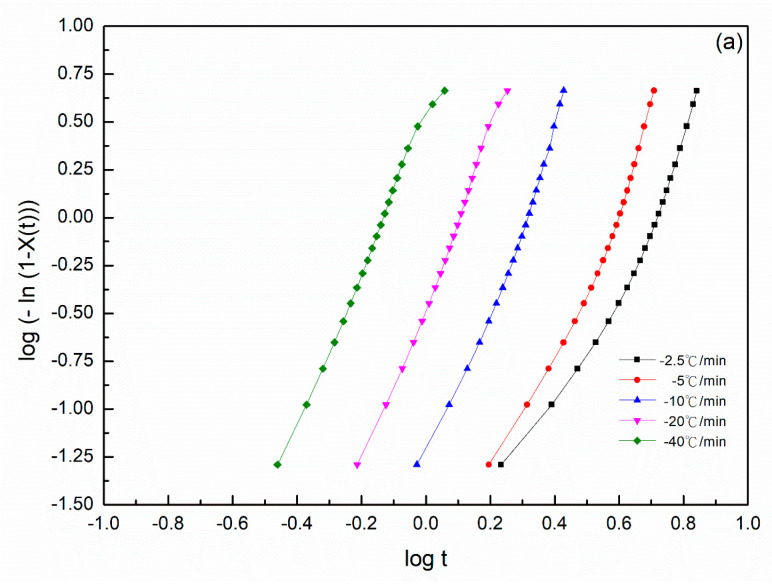

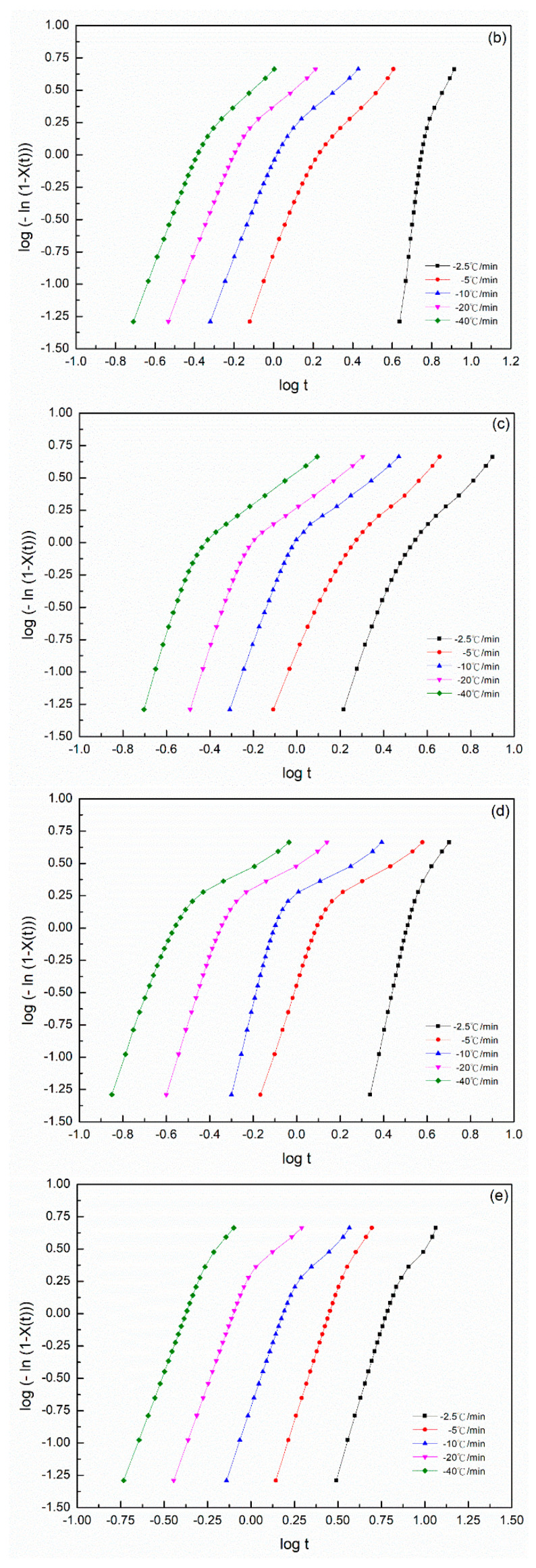
Plots of log{−ln[1−x(t)]} versus logt for the nonisothermal crystallization of (**a**) PA66-BP, (**b**) PA66-NU, (**c**) PA66-FA, (**d**) PA66-HS and (**e**) PA66-MC.

The plotting of log{−ln[1−X(t)]} versus logt (Figure 9) were plotted on the basis of Equations (14) and (15). The values of *n* and $Z_{t}$ or $Z_{c}$ were determined from the slope and intercept of the plots. For the isothermal crystallization process (Figure 1), the values of *n*, $Z_{t}$, $Z_{c}$ and *t*_1/2_ are listed in Table 3 for all the nylon 66 samples at each cooling rate. The curves of all the SP PA66 samples could be divided into two sections, which were the primary crystallization stage and secondary crystallization stage. The curves indicated the existence of a secondary crystallization in the process of nonisothermal crystallization processes for the SP PA66 samples. During the initial primary stages of crystallization, the Avrami exponent *n*_1_ varied from approximately 3.23 to 4.31 for the PA66-FA sample, 4.19 to 7.53 for the PA66-HS sample and 3.63 to 4.36 for the PA66-MC sample, indicating a three-dimensional growth phenomenon [23]. In the primary stage, the Avrami exponent, *n*_1_ was >5, indicating that the nonisothermal crystallization process of the PA66-HS sample was more complicated than the isothermal crystallization process. Compared with the value of *n*_1_, those of *n*_2_ were 1.22–1.66 for the PA66-FA sample, 0.94–3.00 for the PA66-HS sample and 1.34–2.40 for the PA66-MC sample; these values considerably decreased due to spherulite impingement in the later stages of crystallization [38,39]. The Avrami exponent *n*_2_ ≈ 1 (Table 3) was dued to the spherulites’ impingement and crowding. When spherulites exhibited one-dimensional growth, the crystallization mode became simpler [40] at the secondary stage. The $Z_{t}$ values were found to be strongly dependent on the cooling rate and increased with the cooling rate. This finding can be attributed to the fact that at faster cooling rates, crystallization occurred at lower temperatures, thus resulting in faster cooling rates. Nylon 66 has higher water absorption when dissolved in solvent and acted as a chain scission. We believe the solvent acted by virtue of a strong, highly specific polar force to nylon 66. In our study, the sulfuric acid was a strong inorganic acid and regarded as corrosive, while formic acid was a weak organic acid and m-cresol was compatible with nylon 66. Among them, sulfuric acid had the best effect.

#### 3.3.2. Modified Avrami Equation by Ozawa

Considering the effect of *Φ*, Ozawa [41] modified the Avrami equation, Equation (14), into one accounting for all of the processes of nonisothermal crystallization, assuming that the nonisothermal crystallization process is a result of infinitesimally isothermal crystallization procedures, as follows

(16) $$1-X\left(T\right)=exp\left[-K\left(T\right)/\Phi^{m}\right]$$

**Figure 10:**
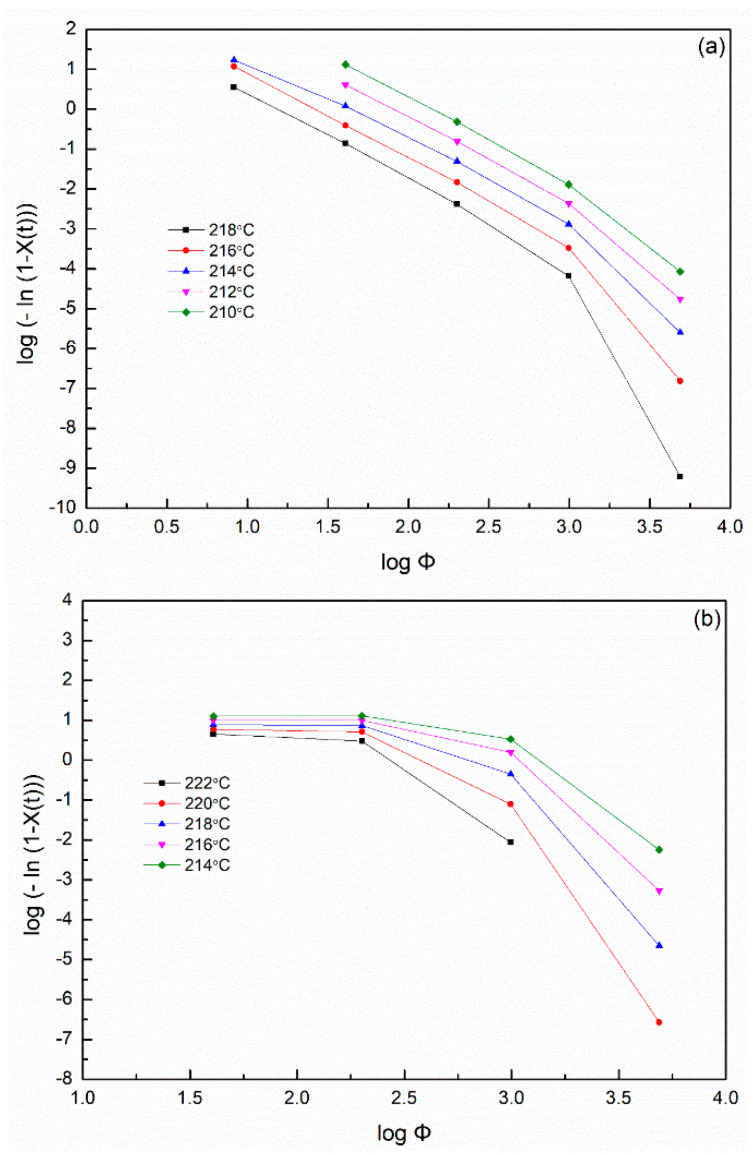

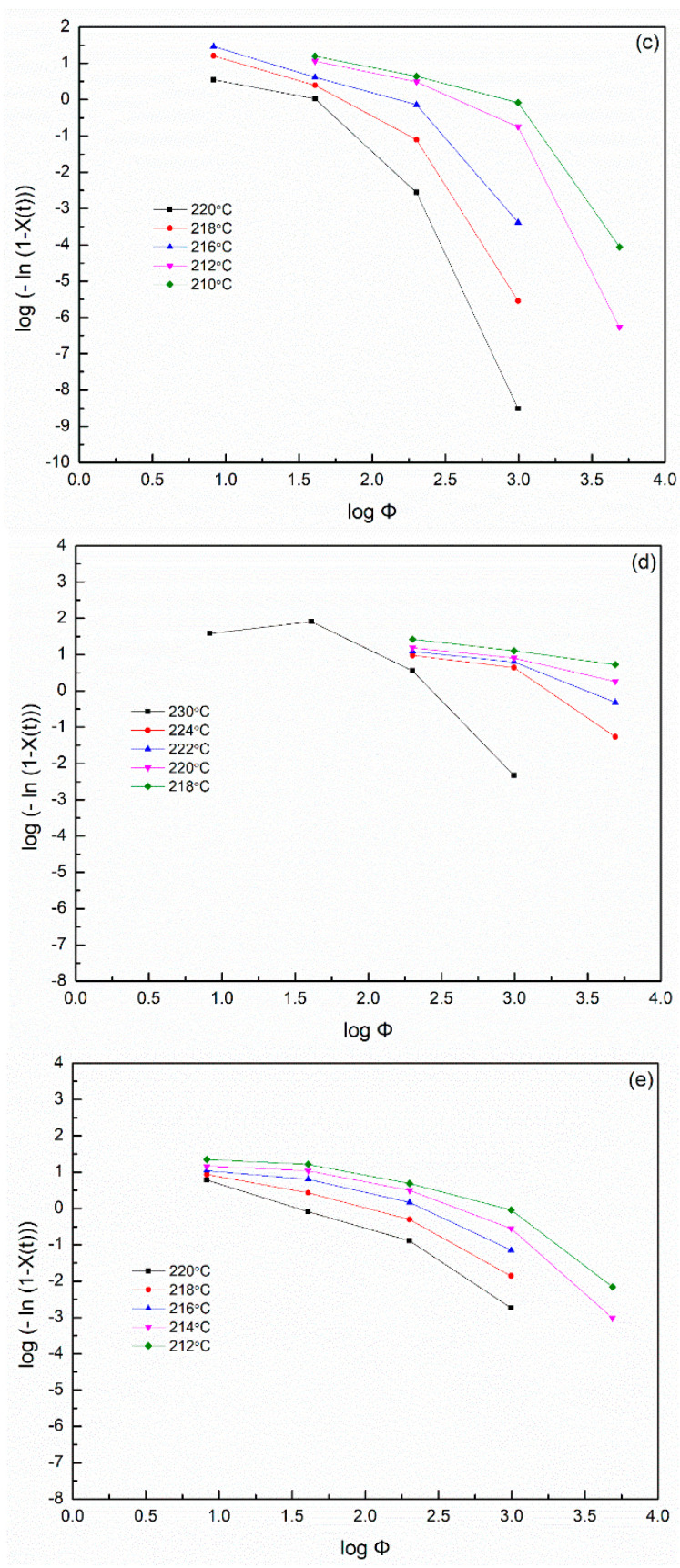
Plots of log{−ln[1−X(t)]} versus logt from the Ozawa equation in the process of nonisothermal crystallization of (**a**) PA66-BP, (**b**) PA66-NU, (**c**) PA66-FA, (**d**) PA66-HS and (**e**) PA66-MC.

(17) log{−ln[1−X(T)]}=−m logΦ+logK(T)

where *X*(*T*) is the relative crystallinity, *K*(*T*) is the kinetics crystallization rate constant and *m* is the Ozawa exponent. If the plotting of logΦ versus log{−ln[1−X(T)]} is in accordance with Equation (17), a series of straight lines should be obtained. The experimental findings indicated that Equation (17) was limited to describing the nonisothermal crystallization kinetics of the SP PA66 (Figure 10). When considered in light of Ozawa’s theory, poor fitting of the equation was observed. The sluggish secondary crystallization and the dependency of lamellar thickness on the temperature were not observed; therefore, the model could not describe the complete nonisothermal process of polymers [42,43,44,45].

#### 3.3.3. The Mo Method

To develop a more appropriate model to depict the nonisothermal crystallization behavior, a convenient approach was adopted by Mo [45,46], combining the Avrami equation with the Ozawa’s equation. The Mo equation could efficiently describe the nonisothermal crystallization and successfully characterized the nonisothermal crystallization behaviors of nylon 11 [38], nylon 12 [39], nylon 66 [30], nylon 69 [47], nylon 46 [48], nylon 1212 [49], nylon1010 [50], nylon composites [51,52,53], polyetherdiphenyletherketoneketone [54] and poly-alkylthiophene [55]. The following equations were obtained:

(18) $$\log\;Z_{t}+n\;\log\;t=\log K\left(T\right)-m\;\log\;\Phi$$

(19) $$\log\Phi =\frac{1}{m}\log\left[K\left(T\right)/Z_{t}\right]-\frac{n}{m}\log t\;$$

Let $F\left(T\right)=\left[K\left(T\right)/Z_{t}\right]{\;}^{1/m}$ and *a* = *n*/*m*; the parameter F(T) refers to the value of the cooling rate chosen at a unit crystallization time when the system has a certain degree of crystallinity. The smaller the value of F(T ) is, the higher the crystallization rate is. F(T) has a definite physical and practical meaning. Through front assumptions, we obtained the following equation:

(20) log Φ=log F(T)−alog t

**Figure 11:**
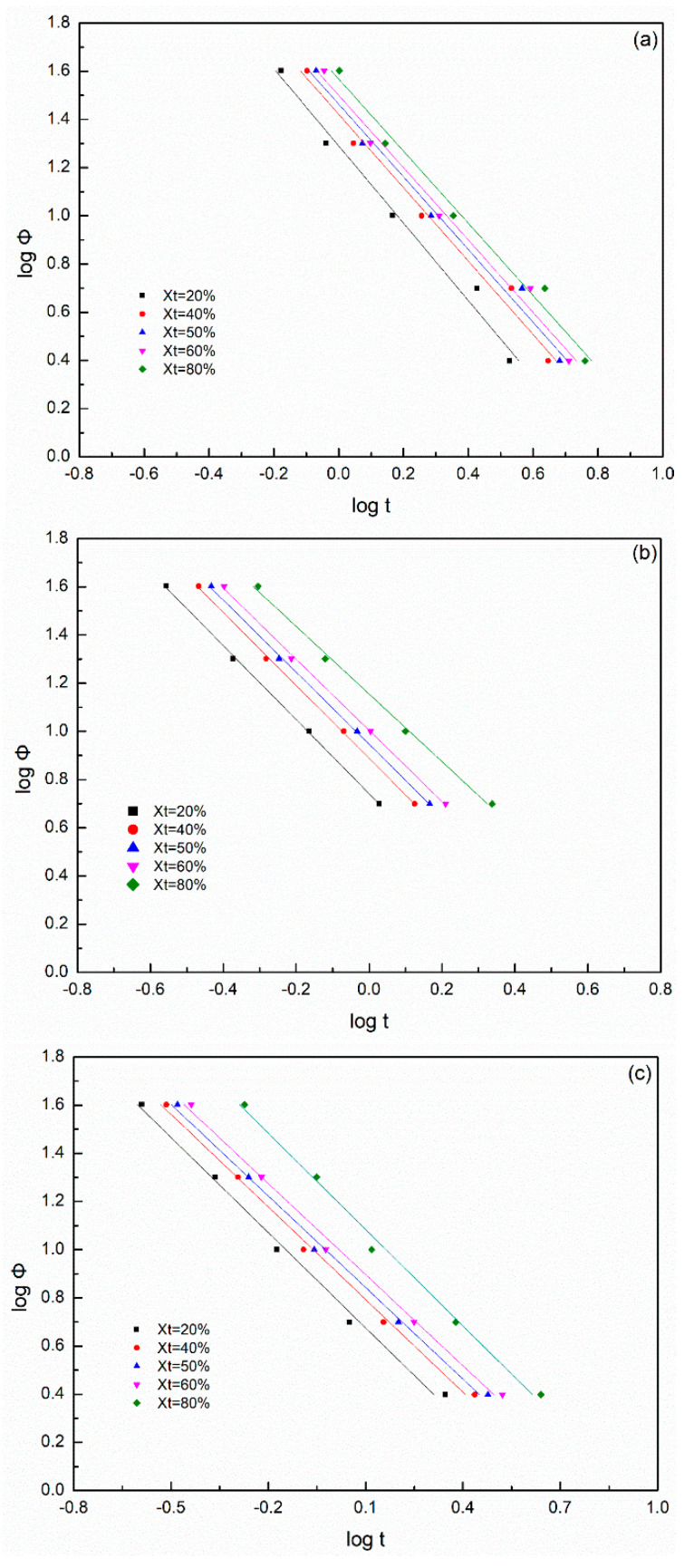

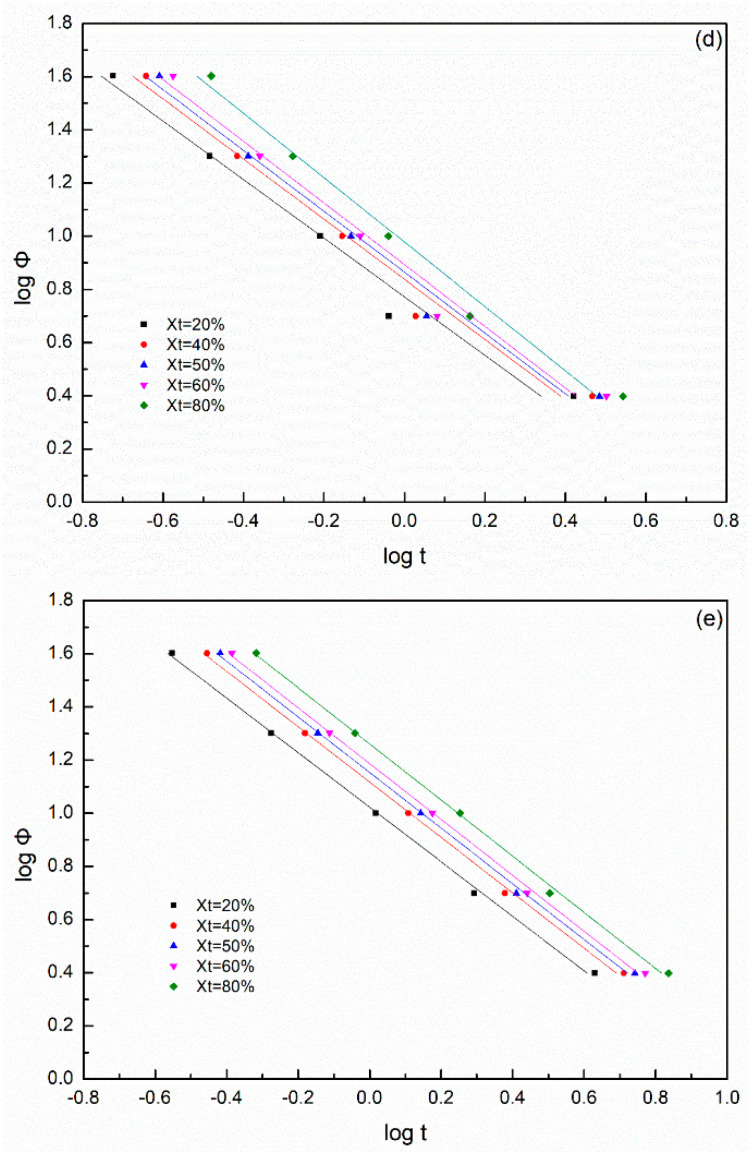
Plots of log Φ versus log t for nonisothermal crystallization of (**a**) PA66-BP, (**b**) PA66-NU, (**c**) PA66-FA, (**d**) PA66-HS and (**e**) PA66-MC at different values of relative crystallinity.

At a given degree of crystallinity of the SP PA66 sample, the plot of log Φ versus log t according to Equation (20) is shown in Figure 11. A linear relationship was noted between log Φ and log t; Mo’s model could accurately account for the nonisothermal crystallization kinetics of the SP PA66 sample. When the cooling rate was low (Φ= 2.5 °C min^−1^), the crystallization time *t* was extended, resulting in the impingement of spherulites in the later stage of PA66-HS [42]. By using a straight line to fit these data points, we obtained straight lines with slope = −*a* and intercept = log F(T). As shown in Table 4, with the increase in relative crystallinity, the values of F(T) increased. At a set crystallization time, a faster cooling rate was required to obtain a higher crystallinity. The values of *a* remained almost constant during the crystallization of each sample, indicating that the style of crystallization did not change during crystallization. By contrast, a change in crystallization was observed depending on the solvent, suggesting that the solvent affected the style of crystallization of SP PA66. At a set relative crystallinity, the F(T) values for the SP PA66 and PA66-NU samples were lower than that for the neat nylon PA66-BP sample, and the value of PA66-HS was the lowest. These findings indicated that nylon 66 with a nucleating agent and SP PA66 had a faster crystallization rate than the neat nylon PA66-BP, and nylon PA66-HS had a faster crystallization rate than PA66-MC and PA66-FA. This result is consistent with the result of the Jeziorny method.

### 3.4. Crystallization Activation Energy *Δ*E

**Figure 12:**
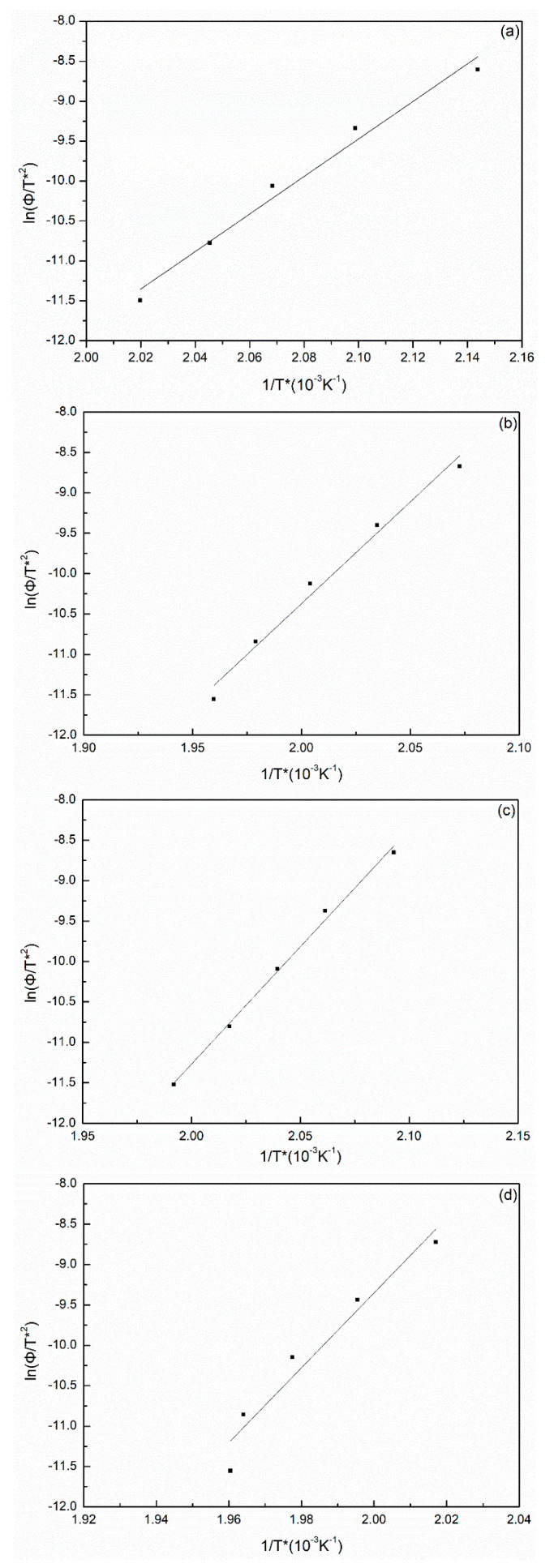

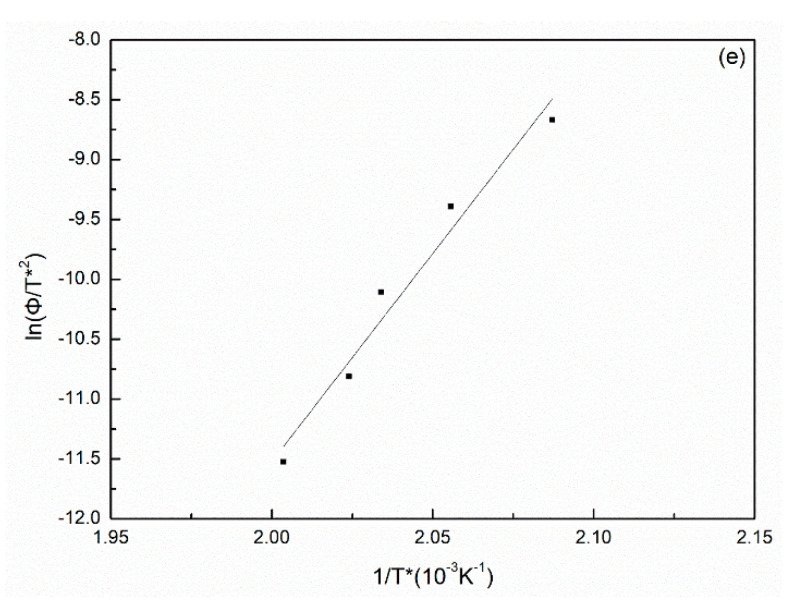
The plots of $\ln\;\Phi /T^{*2}$ versus $1/T^{*}$ from the Kissinger method of (**a**) PA66-BP, (**b**) PA66-NU, (**c**) PA66-FA, (**d**) PA66-HS and (**e**) PA66-MC.

Activation energy is a crucial parameter in the phase transition process and was controlled by two factors: one was related to the transport of crystalline units across the phase and the other was related to the free energy barriers for nucleation [56]. Activation energy can effectively reflect the crystallization ability of polymers. Considering the effect of T* with the cooling rate Φ in the nonisothermal crystallization process, Kissinger [57] reported that the activation energy Δ*E* of nonisothermal crystallization can be determined as follows

(21) $$\frac{d\left[\ln\left(\frac{\Phi }{T^{*2}}\right)\right]}{d\left(\frac{1}{T^{*}}\right)}=-\frac{\Delta E}{R}\;$$

where *R* is the gas constant and T* is the peak temperature. We obtained a straight line in the plot of $\ln\left[\Phi /T^{*2}\right]$ versus 1/T*, indicating a linear relationship (Figure 12). The activation energy Δ*E* could be derived from the slope of the plots. ΔE = −245, −385 and −289 kJ/mol for the PA66-FA, PA66-HS and PA66-MC samples, respectively. Moreover, the Δ*E* value of the PA66-BP was −449 kJ/mol in our experiment. All the Δ*E* values of SP PA66 were lower than those of PA66-BP. Hence, the SP PA66 sample had a faster crystallization rate than PA66-BP.

### 3.5. Crystallite Morphology

**Figure 13:**
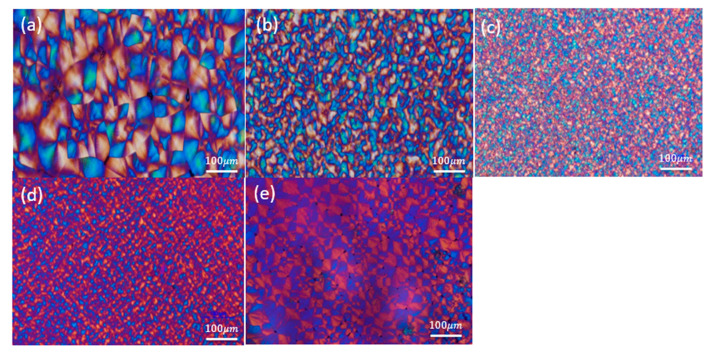
Polarized optical micrographs for solvent-precipitated nylon 66 anneals at 300 °C for 30 min and slow cooled off (**a**) PA66-BP, (**b**) PA66-NU, (**c**) PA66-FA, (**d**) PA66-HS and (**e**) PA66-MC.

**Figure 14:**
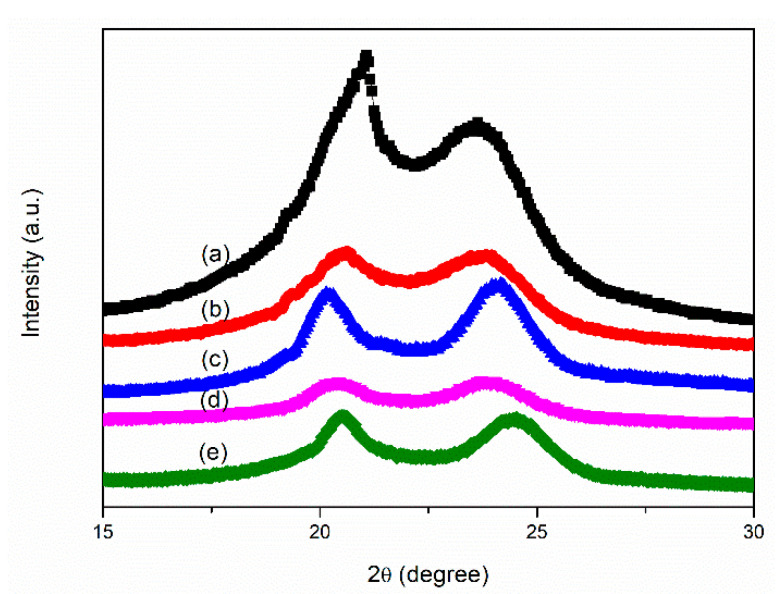
WAXS scans for solvent-precipitated (**a**) PA66-BP, (**b**) PA66-NU, (**c**) PA66-FA, (**d**) PA66-HS and (**e**) PA66-MC.

POM is one of the predominant and most informative tools used for investigating spherulitic morphologies [58,59,60,61]. Figure 13 presents a series of POM micrographs of nylon 66 samples obtained after melting them at 300 °C, annealing for 10 min and then quenching to *T*_c_ = 230 °C. The spherulites were clearly negative in view of the characteristic of the Maltese cross. For the neat polymer PA66-BP, large impinged spherulites with well-defined Maltese cross spherulites were observed (Figure 13a). The micrograph exhibited Maltese cross spherulites, as shown (Figure 13b–e) for the SP PA66 samples; these spherulites were smaller due to the increasing nucleation density. The general size of the spherulites within the PA66-HS sample (Figure 14d) was more uniform than those within the PA66-FA and PA66-MC samples (Figure 14c,e). The morphology of the PA66-HS sample had the smallest structure.

### 3.6. Characterization of Solvent-Precipitated Nylon 66

Most even-even nylon exhibited the α form of crystals at room temperature. Bunn and Garner reported that nylon 66 and nylon 610 have triclinic structures consisting of fully extended chains joined by hydrogen bonds and exhibit two characteristic peaks in their X-ray diffractograms at room temperature [62]. XRD patterns (Figure 14) were used to investigate the crystallization of SP PA66; Figure 14a shows a single broad peak with the maximum peak at 2θ = 21.0° and 23.8° for the PA66-BP sample, which is similar to the diffraction peaks of the nylon 66 α phase reported by Murthy and co-workers [63]. This maximum peak α_1_ corresponded to ordering along the (010) plane and is attributable to the distance between the hydrogen-bonded chains. The second maximum peak α_2_ results from ordering along the (010) and (110) planes and is attributed to the separation between hydrogen-bonded sheets. The XRD scan for PA66-NU is shown in Figure 14b. Two maximum peaks were located at 2θ = 20.6° and 23.8°, which are similar to that of PA66-BP. The XRD scan for PA66-FA shown in Figure 14c is similar to that of PA66-BP and PA66-NU. Two distinguishable peaks were evident, with the maximum 2θ = 20.1° and 24.2°. XRD scans for the PA66-HS and PA66-MC samples shown in Figure 14(d),(e), respectively, are similar to those for PA66-FA. The maximum peaks were located at 2θ = 20.3° and 23.8° for PA66-HS and 20.1° and 24.5° for PA66-MC. These results indicated that the SP PA66 did not change the crystal structures of neat nylon 66. According to the XRD scan shown in Figure 14, the d-spacing of the (100) plane of nylon 66 treated with a nucleating agent and a solvent became wider, and the d-spacing of the (010/110) plane became narrower. This phenomenon was caused by the combination of the characteristics of triclinic crystals and hydrogen bonding. The SP PA66 samples had high crystallinity and a perfect crystal structure [64]. Two strong and separated diffraction peaks were observed (Figure 14c–e). Therefore, SP PA66 had a perfect structure, which neat PA66-BP did not.

## 4. Conclusions

First of all, the precipitation process that we have studied may readily be called the crystal growth enhanced by fluctuations. As can be seen from POM, it leads rapidly to the formation of many “induced nuclei sites” due to the reordering of the hydrogen bond inside the SP PA66 system. This study investigated the isothermal and nonisothermal crystallization kinetics of SP PA66 through DSC. The results indicated that the isothermal crystallization kinetics followed the Avrami behavior during isothermal crystallization from the melting stage. The Avrami exponent n was between 2 and 3, indicating a two- and three-dimensional processes. The different theories of Jeziorny, Ozawa and Mo were examined to study the nonisothermal crystallization kinetics of SP PA66. The results of the Jeziorny equation analysis revealed that the nonisothermal crystallization could be a two-stage process for SP PA66. At the primary stage, the n_1_ value ranged from 3 to 7, corresponding to a three-dimensional and complicated mechanism. The n_2_ value ranged from 1 to 3 in the secondary stage. The reduction in the value of n_2_ compared with that of n_1_ was due to the spherulite impingement and crowding. The Ozawa equation could not describe the nonisothermal crystallization kinetics of SP PA66. The Mo equation successfully described the two stages of the nonisothermal crystallization process of SP PA66. The values of F(T) were strongly rate dependent on the crystallization in the case of SP PA66 and lower than those of the neat nylon 66 at the same relative crystallinity. In addition, the XRD and POM results indicated that SP PA66 significantly decreased the size of spherulites and had an increased spherulite number; no change in the crystal form was noted. The crystallization rate of SP PA66 compared with neat nylon 66 was increased.

## Figures and Tables

**Table 1:** Avrami parameters *n*, *k*, $t_{max}$, $t_{1/2}$ and $\tau_{1/2}$ from the Avrami equation.

Sample	*T_c_* (°C)	*n*	*K*(min^−1^)	*t_max_* (min)	*t*_1/2_ (min)	*τ*_1/2_ (min^−1^)	*X*(*t_max_*) (%)
PA66-BP	219	2.406	0.782	1.267	1.360	0.732	41.83
	221	2.490	0.402	1.700	1.706	0.586	49.60
	223	2.730	0.160	2.150	2.380	0.447	45.52
	225	2.813	0.052	3.067	3.116	0.321	48.13
	227	2.838	0.016	4.333	4.356	0.230	49.42
PA66-NU	219	2.341	10.35	0.467	0.529	1.890	34.50
	221	2.299	7.963	0.517	0.576	1.736	36.81
	223	2.403	6.126	0.567	0.632	1.582	37.00
	225	2.374	4.206	0.633	0.712	1.405	36.55
	227	2.338	3.061	0.717	0.808	1.238	36.52
PA66-FA	219	2.551	140.8	0.100	0.124	8.065	33.57
	221	2.477	78.67	0.167	0.181	5.525	42.22
	223	2.255	22.25	0.400	0.431	2.320	38.93
	227	2.228	9.343	0.433	0.508	1.969	32.00
	229	2.236	3.017	0.633	0.761	1.314	31.52
PA66-HS	221	2.888	355.3	0.200	0.215	4.651	29.98
	223	2.952	94.45	0.300	0.321	3.115	29.44
	227	2.654	25.35	0.467	0.490	2.041	42.33
	229	2.751	3.030	0.800	0.882	1.134	37.66
	231	2.842	0.340	1.450	1.675	0.597	34.28
PA66-MC	219	2.359	12.91	0.417	0.452	2.212	39.06
	221	2.416	4.996	0.467	0.507	1.972	39.47
	223	2.277	1.968	0.500	0.553	1.808	37.96
	227	2.122	1.045	0.567	0.628	1.592	38.89
	229	2.141	1.968	0.767	0.882	1.134	36.63

**Table 2:** The values of T*, $t_{max}$ and *X(t)* in nonisothermal crystallization.

Sample	*Φ* (°C/min)	T* (°C)	*t_max_* (min)	*X*(*t*) (%)
PA66-BP	2.5	221.95	5.22	61.71
	5	215.75	4.10	69.06
	10	210.33	2.12	66.76
	20	203.32	1.26	61.40
	40	193.30	0.72	59.22
PA66-NU	2.5	237.12	5.17	57.12
	5	232.16	1.30	33.03
	10	225.83	0.89	43.95
	20	218.32	0.55	46.63
	40	209.30	0.35	43.21
PA66-FA	2.5	228.87	2.47	29.34
	5	222.50	1.37	35.81
	10	217.16	0.81	41.11
	20	211.98	0.50	37.90
	40	204.63	0.30	37.87
PA66-HS	2.5	236.95	3.05	50.00
	5	236.01	1.10	44.20
	10	232.53	0.70	39.21
	20	227.98	0.38	39.81
	40	222.63	0.23	42.20
PA66-MC	2.5	225.95	5.45	48.19
	5	220.91	2.59	50.22
	10	218.49	1.37	47.64
	20	213.32	0.72	50.00
	40	205.97	0.35	50.33

**Table 3:** The values of $n,\;Z_{t}\;and\;Z_{c}$ from the Avrami equation at the two stages of nonisothermal crystallization.

		Primary Crystallization Stage			Secondary Crystallization Stage		
Sample	Φ (°C/min)	*n*_1_	*Z_t_*_1_	*Z_c_*_1_	*n*_2_	*Z_t_*_2_	*Z_c_*_2_
PA66-BP	2.5	3.012	0.006	0.130	5.520	0.0001	0.025
	5	3.449	0.008	0.379	6.426	0.0001	0.167
	10	4.036	0.049	0.740	6.221	0.009	0.627
	20	4.306	0.335	0.947	4.759	0.344	0.948
	40	4.130	3.387	1.031	3.499	3.474	1.032
PA66-NU	2.5	3.202	0.005	0.121	1.663	0.058	0.321
	5	3.518	0.176	0.706	1.587	0.464	0.858
	10	3.723	0.906	0.990	1.329	1.215	1.020
	20	3.848	6.124	1.095	1.307	2.352	1.044
	40	3.941	34.74	1.093	1.453	4.541	1.039
PA66-FA	2.5	3.690	0.017	0.169	1.660	0.137	0.451
	5	3.232	0.151	0.686	1.563	0.401	0.833
	10	4.196	1.185	1.017	1.258	1.126	1.012
	20	4.306	8.644	1.114	1.250	1.854	1.031
	40	4.226	69.167	1.112	1.225	3.484	1.032
PA66-HS	2.5	7.531	0.015	0.030	3.007	0.039	0.274
	5	5.044	0.355	0.813	1.005	1.012	1.003
	10	6.227	4.465	1.161	0.943	1.803	1.061
	20	4.922	54.06	1.221	0.983	3.111	1.058
	40	4.187	235.2	1.146	0.964	4.672	1.039
PA66-MC	2.5	4.358	0.001	0.044	1.760	0.056	0.316
	5	4.149	0.014	0.424	2.402	0.104	0.635
	10	3.881	0.191	0.848	1.338	0.765	0.974
	20	3.749	2.416	1.050	1.345	1.969	1.035
	40	3.633	22.91	1.080	2.211	8.482	1.055

**Table 4:** The values of a and F(T) at a certain degree of crystallinity from Mo method.

Sample	*Xt* (%)	*a*	*F*(*T*)
PA66-BP	20	1.284	17.28
	40	1.251	22.39
	50	1.246	24.34
	60	1.246	26.19
	80	1.250	30.10
PA66-NU	20	1.507	5.536
	40	1.489	7.716
	50	1.474	8.802
	60	1.455	10.04
	80	1.383	14.16
PA66-FA	20	1.292	6.390
	40	1.252	9.266
	50	1.245	10.45
	60	1.264	12.39
	80	1.351	24.09
PA66-HS	20	1.282	4.820
	40	1.320	6.023
	50	1.336	6.694
	60	1.354	7.556
	80	1.389	11.08
PA66-MC	20	1.025	10.53
	40	1.040	13.10
	50	1.046	14.23
	60	1.050	15.36
	80	1.055	18.21

## Data Availability

The data presented in this study are available on request from the corresponding author.
